# Supplementary material for: Identifying zooplankton community changes between shallow and upper-mesophotic reefs on the Mesoamerican Barrier Reef, Caribbean
Source: PeerJ. 2017 Jan 5;5:e2853. doi: 10.7717/peerj.2853 (PMC5289443; doi:10.7717/peerj.2853)
Supplement: Table S1 [file peerj-05-2853-s001.docx]

Supplemental Table S1. GPS locations of lionfish abundance survey locations around Utila, Honduras. All GPS coordinates are given in WGS 84.

| Survey Site | Latitude | Longitude |
| --- | --- | --- |
| Little Bight | 16.07926302 | -86.92942222 |
| Black Coral Wall | 16.08305968 | -86.91699554 |
| Coral View | 16.08823274 | -86.91094506 |
